# Hepatoprotective and anti-obesity effects of Korean blue honeysuckle extracts in high fat diet-fed mice

**DOI:** 10.20463/jenb.2018.0029

**Published:** 2018-12-31

**Authors:** Yoon-Seok Chun, Se-Kwang Ku, Jong-Kyu Kim, Sok Park, In-ho Cho, Nam-Ju Lee

**Affiliations:** 1 Aribio Central Research Institute, Pangyo Republic of Korea; 2 Department of Anatomy and Histology, Daegu Haany University, Gyeongsan Republic of Korea; 3 Kwangwoon University, Seoul Republic of Korea; 4 Human Performance Laboratory, Korea National Sport University, Seoul Republic of Korea; 5 Department of Leisure Sports, Jungwon University, Goesan-gun Republic of Korea

**Keywords:** Metformin, Berries of Lonicera Caerulea L., non-alcoholic fatty liver disease, AMPK (5' adenosine monophosphate-activated protein kinase)

## Abstract

**[Purpose]:**

This study aimed to study the protective effects and mechanism of Blue Honeysuckle (BH) extracts (Berries of Lonicera caerulea L.) on non-alcoholic fatty liver disease (NAFLD) and obesity risk factors in a high fat-diet (HFD) model.

**[Methods]:**

Animals adapted to HFD were selected after 1 week of adaption period and divided into 6 groups (8 mice in each group; 40 HFD-fed mice and 8 normal fat pellet diet (NFD)-fed mice). After the end of 12 weeks of continuous oral administrations of 3 different dosages of BH extract, 400, 200 and 100 mg/kg, or metformin 250 mg/kg, dissolved in a volume of 10 mL/kg distilled water, the hepatoprotective, hypolipidemic, hypoglycemic, nephroprotective, and anti-obesity effects were analyzed.

**[Results]:**

The BH extract improved fat density and mass, adipocyte histopathology, hepatocyte hypertrophy, hepatic enzyme activity, lipid metabolism, and related gene expression including ACC1, AMPK α1 and AMPK α2 in hepatic tissue, leptin, UCP2, adiponectin, C/EBP α, C/EBPβ and SREBP1c in adipose tissue. Especially, 200 mg/kg of BH extract constantly improved NAFLD and obesity risk factors through AMPK upregulation-mediated hepatic glucose enzyme activity, lipid metabolism-related gene expression, and activation of the antioxidant defense system, to a level comparable to that of metformin 250 mg/kg in HFD-fed mice.

**[Conclusion]:**

BH extract has the potential to reduce the risk factors associated with obesity, in addition to the remarkable effect of preventing NAFLD. Future research will need to be done to determine whether these results are consistent in human studies.

## INTRODUCTION

In the past 30 years, the incidence of non-alcoholic fatty liver disease (NAFLD) has been increasing owing to changes in dietary habits and Western lifestyles in the Asia-Pacific region^1^. Liver damage by NAFLD causes hepatic metabolic dysfunction. NAFLD is the most common chronic liver disease and is associated with an increase in serum alanine transaminase, as well as disorders such as benign macrovesicular hepatosteatosis, non-alcoholic steatohepatitis, hepatic fibrosis, cirrhosis of the liver, and hepatocellular carcinoma^2^; moreover, increased triglyceride levels are associated with these tissue changes. In particular, the accumulated fat in NAFLD can increase oxidative stress^3^ in the liver and cause chronic damage to it^4^. 

There is a growing interest in berries as a food for reducing oxidative stress caused by reactive oxygen species because berries have both antioxidant vitamins and enzymes such as superoxide dismutase, glutathione peroxidase, and glutathione reductase, as well as phytochemicals with distinct flavors, odors, and colors and antioxidant functions at the same time^5^. Blue honeysuckle (BH, Lonicera caerulea L.) is known to contain higher vitamin C, total phenolic content, and total anthocyanin content than tomato, bilberry, sea-buckthorn, black currant and Siberian rhubarb^6,7^ and to prevent liver damage^8-10^. BH (L. cearulea) has been domesticated from 1913; however, an earlier example was reported in 1894 in a horticulture plant in Russia^11^. BH is known as haskap or hasukappu in Japan and zhimolost in Russia^7^. 

Wu and colleagues^9^ evaluated the effects of BH extract on high-fat dietary nonalcoholic liver injury in a mouse model after 45 days of BH extract feeding. They determined the effects of BH on the nuclear factor (erythroid-derived 2) -like 2 (Nrf2) and manganese-dependent superoxide dismutase (MnSOD) up-regulation in nonalcoholic steatohepatitis. However, in the BH extract manufacturing process, Chinese HB extract extracted using 75% of ethanol is limited to use as food. Recently, a high-fat diet treated mouse model was used to directly compare the NAFLD improvement of the AMPK activator Metformin and mild obesity by taking BH extract for 12 weeks. As a result, BH extract treatment showed an improvement of NAFLD by ingesting 400 mg/kg BH extract, which is similar to Metformin treatment^10^. However, the consumption of 400 mg/kg needed in the mouse model is not economically feasible due to the consumption of at least 2 g/day on a human basis. The pectinase enzyme used in the manufacturing process of BH extract described by Kim and colleagues^10^ is also used in the production of animal feeds and is restricted to intake as actual food. BH is known to be harvested in Russia, northeastern Asia (China, Japan, and Korea), but in in vitro studies have reported that the antioxidant activity of BH harvested in Korea is superior to that of BH harvested in China^12^. If the economic efficiency and systematicity of the manufacturing process are established in accordance with the ameliorating effect using Chinese BH has on NAFLD^10^, a method of using BH as a NAFLD preventive food will be provided. 

Therefore, the present study aims to confirm the ameliorating effect using BH grown in Korea has on NAFLD by feeding optimized BH extract which can be consumed as normal food to high-fat diet-fed mice.

## METHODS

### Animals and husbandry

**Figure 1.**
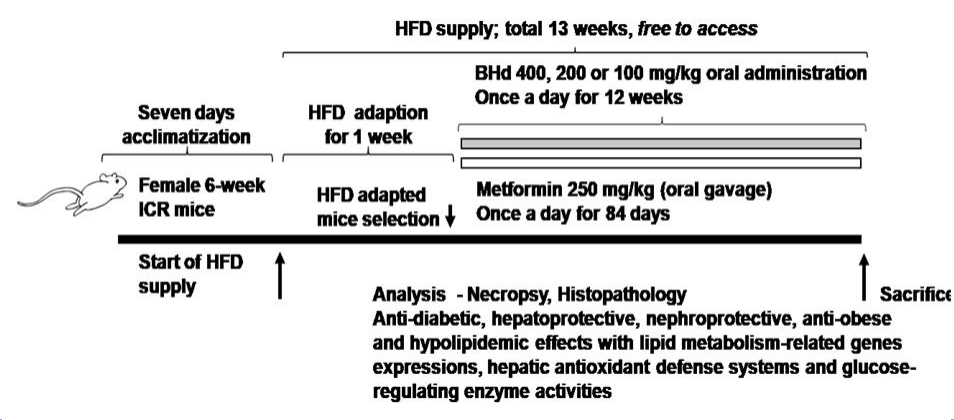
Experimental design of this study.

A total of one hundred female SPF/VAF CrljOri:CD1[ICR] mice (6-wk old upon receipt; Orient Bio, Sungnam, Korea) were used after acclimatization for 7 days. Animals were allocated 4 to 5 per polycarbonate cage in a controlled room under temperature set at 20-25°C and humidity set at 40-45%. The light/dark cycle was 12 hrs, and standard rodent chow (Cat. No. 38057; Purinafeed, Seungnam, Korea) and water were supplied free to access. Adapted animals to High Fat Diet (HFD) were selected at 1 week of adaption period as 6 groups (eight mice in each group, 40 HFD-fed mice, 8 normal diet-fed mice and 8 Metformin mice): 1) Healthy control: 10 mL/kg of distilled water were orally administered to mice with NFD supply, 2) HFD control: 10 mL/kg of distilled water were orally administered to mice with HFD supply, 3) Metformin: 250 mg/kg of metformin were orally administered to mice with HFD supply, 4) BH extract 400: 400 mg/kg of BH extract were orally administered to mice with HFD supply, 5) BH extract 200: 200 mg/kg of BH extract were orally administered to mice with HFD supply, 6) BH extract 100: 100 mg/kg of BH extract were orally administered to mice with HFD supply (Fig.1). All laboratory animals were treated according to the national regulations of the usage and welfare of laboratory animals and approved by the Institutional Animal Care and Use Committee in Daegu Hany University (Gyeongsan, Gyeongbuk, Korea) prior to animal experimentation. 

### Preparation and administration of test substances

BH extract was prepared and supplied by Aribio Co. Ltd. (Seongnam, Korea) as a red colored powder and stored at 4°C in a refrigerator to protect from light and humidity until use. After grinding the raw material of the blue honeysurckle berries (Lonicera caerulea L.), 5 times of water is added to the raw material, and extraction is carried out at room temperature 25°C for 3-5 hours. The extraction was squeezed and filtered, followed by centrifugation at 12,000 rpm using a high-speed centrifuge, and then lyophilized at 55-65°C after concentration (10 brix). The BH extract used in this study contained 1.7% of Cyanidin-3-O-glucoside (C3G), which was determined by high performance liquid chromatography analysis. White powders of metformin hydrochloride (Wako, Osaka, Japan) were used as reference recommendation drugs as follows. Appropriate amounts of BH extract were dissolved in distilled water as 40, 20 and 10 mg/mL concentration, and were orally administered once a day for 84 days in a volume of 10 mL/kg (equivalent to 400, 200 and 100 mg/kg), using a stainless zonde attached to 1 mL syringe , from 1 weeks after initial HFD supply. In addition, metformin HCl was dissolved in distilled water in a concentration of 25 mg/mL and also orally administered in a volume of 10 mL/kg, equivalent to 250 mg/kg, once a day for 84 days from 1 week after initial HFD supply. In the intact vehicle and HFD control mice, equal volumes of distilled water were also orally administered, instead of test substances to provide the same restraint stresses caused by gastric gavages. The dosage of BH extract was selected as 400, 200 and 100 mg/kg considering the results of our previous studies^9,10^, and administration volume was decided to 10 mL/kg, the general dosing volume in mice [KFDA Guidelines, Notification No. 2015- 082, 2015], in the current experiment. The dose levels of metformin, 250 mg/kg were also selected based on our previous studies^13,14^.

### Body and organ weight change

Changes of body weight were measured at 8 days (immediately before the start of HFD supply), 1 day before initiation of administration, on the initial administration day and then weekly until termination of the study. The weight was determined by using an automatic electronic balance (XB320M, Precisa Instrument, Zuerich, Switzerland). At initiation and at termination of administration, all experimental animals were fasted overnight (water was not withheld; about 12 hrs) to reduce the differences from feeding. The fasting before we started the experiment was to maximize the effect of feeding with feed, and the fasting on the last day of the experiment was due to the dissection to confirm the effects of the drug administration. In addition, body weight gains were additionally calculated during adaptive periods (from Day -8 to Day 0 of test article-administration) and administration periods (from Day 0 to Day 84 of test article-administration).

### Mean daily food consumption

Diets (150 g in each individual cage) were supplied, and remainder amounts of supplied diets were measured at 24 hours by using an automatic electronic balance (XB320M, Precisa Instrument, Zuerich, Switzerland). The measured daily food consumption was then divided by reared animal numbers in the same cages. These were regarded as the individual mean daily food consumption of mice (g/day/mice) and calculated as [Amounts of supplied diets (150 g) – Amounts of remainder supplied diets after 24 hours]/reared head of mice. These measurements were conducted once a week in the 84 days of administration periods^13,14^.

### Body fat and abdominal fat density

The mean fat densities of the total body and abdominal cavity regions of each mouse were detected by in live DEXA (InAlyzer; Medikors, Seungnam, Korea) once at the end of the 84 days of continuous treatment with test material administration.

### Serum biochemistry analyses

Some collected blood samples from the vena cava at 84 days after initial test substance treatment under inhalation anesthesia, were deposited into clotting activated serum tubes, and centrifuged at 15,000 rpm for 10 minutes at room temperature. Serum aspartate transaminase (AST), alanine aminotransferase (ALT), alkaline phosphatase (ALP), lactate dehydrogenase (LDH), γ-glutamyltransferase (GGT), creatinine, and cholesterol family levels were measured using an automated blood analyzer (Dri-Chem NX500i; Fuji Medical System Co., Ltd., Tokyo, Japan), after stored in an ultra deep freezer (MDF-1156, Sanyo, Tokyo, Japan) at -150ºC.

### Measurement of lipid compositions in the feces

Lipids were extracted from the feces collected at 8 hours after the last test substance administration, according to the method of Folch et al.^16^. The concentrations of fecal total cholesterol (TC) and triglycerides (TG) were measured by a colorimetric assay using a commercial kit (TC colorimetric assay kit, Cat. No. 100102303, Cayman, Ann Arbor, MI, USA; Total Cholesterol Assay Kit (Colorimetric), Cat. No. STA-384, Cell Biolabs, San Diego, CA, USA) using a microplate reader (Sunrise, Tecan, Männedorf, Switzerland).

### Liver lipid peroxidation and antioxidant defense system

After measurements of organ weights, the MDA and GSH contents and the CAT and SOD enzyme activities in mouse hepatic tissues were assessed. Separated hepatic tissues were weighed and homogenized in ice-cold 0.01 M Tris-HCl (pH 7.4) using a bead beater (TacoTMPre, GeneResearch Biotechnology Corp., Taichung, Taiwan) and an ultrasonic cell disruptor (KS-750, Madell Technology Corp., Ontario, CA, USA), and then centrifuged at 12,000 × g for 15 minutes as described by Kavutcu et al.^16^. Tissue homogenates were stored in an ultra deep freezer at -150ºC until analysis. The concentration of liver lipid peroxidation was determined by estimating MDA using the thiobarbituric acid test and a UV/Vis spectrophotometer (OPTIZEN POP, Mecasys, Daejeon, Korea) at absorbance 525 nm, as nM of MDA/mg protein [Jamall and Smith, 1985] . The content of total protein was measured by using a previously described method^17^ using bovine serum albumin (Invitrogen, Carlsbad, CA, USA) as an internal standard. Prepared hepatic homogenates were mixed with 0.1 mL of 25% trichloroacetic acid (Merck, West Point, CA, USA), and then centrifuged at 4,200 rpm for 40 min at 4 ºC. GSH contents were spectrophotometrically measured at an absorbance of 412 nm using 2-nitrobenzoic acid (Sigma-Aldrich, St. Louise, MO, USA). Decomposition of H_2_O_2_ in the presence of CAT was detected at 240 nm using a spectrophotometer. CAT activity was defined as the amount of enzyme required to decompose 1 nM of H_2_O_2_ per minute at 25 °C and pH 7.8. Results were expressed as U/mg protein. Measurements of SOD activities were made according to Sun et al.^18^. SOD estimation was based on the generation of superoxide radicals produced by xanthine and xanthine oxidase, which react with nitrotetrazolium blue to form a formazan dye. SOD activity was then spectrophotometrically measured at 560 nm as the degree of inhibition of this reaction and expressed as U/mg protein. One unit of SOD enzymatic activity is equal to the amount of enzyme that diminishes the initial absorbance of nitrotetrazolium blue by 50% during 1 min.

### Analysis of hepatic glucose-regulating enzyme activity

Hepatic tissue weighing 0.3 g was homogenized in buffer solution (0.1 M triethanolamine, 0.2 M EDTA, and 0.002 M dithiothreitol) and centrifuged at 1,000 × g for 15 min at 4 °C. The supernatant was further centrifuged at 10,000 × g for 15 min at 4 °C. The GK activity was measured according to the method described by Davidson and Arion [1987] with slight modifications. 0.98 mL of the reaction mixture (50 mM Hepes-NaGT (pH 7.4), 100 mM KCl, 7.5 mM MgCl_2_, 2.5 mM dithioerythritol, 10 mg/mL albumin, 10 mM glucose, 4 units of glucose-6-phosphate dehydrogenase, 50 mM NAD+, and 10 μl hepatic tissue homogenates) was pre-incubated at 37 °C for 10 min. The reaction was initiated with the addition of 10 μl of 5 mM ATP and the mixture was incubated at 37 °C for 10 min. The change in absorbance at 340 nm was recorded. The G6pase activity was measured based on the method of Alegre et al. [1988] . The reaction mixture contained 765 μl of 131.58 mM Hepes-NaGT (pH 6.5), 100 μl of 18 mM EDTA (pH 6.5), 100 μl of 265 mM glucose-6-phosphate, 10 μl of 0.2 M NADP+, 0.6 IU/mL mutarotase, and 0.6 IU/mL glucose dehydrogenase. After pre-incubation at 37 °C for 3 min, the mixture was added to 5 μl hepatic tissue homogenates and incubated at 37 °C for 4 min. The change in absorbance at 340 nm was measured. The PEPCK activity was measured using the method of Bentle and Lardy [1976] . The reaction mixture contained 72.92 mM sodium Hepes (pH 7.0), 10 mM dithiothreitol, 500 mM NaHCO_3_, 10 mM MnCl_2_, 25 mM NADH, 100 mM IDP, 200 mM PEP, 7.2 units of malic dehydrogenase, and 10 μl of hepatic tissue homogenate. The enzyme activity was determined based on the decrease in the absorbance of the mixture at 340 nm at 25 °C using a UV/Vis spectrophotometer (OPTIZEN POP, Mecasys, Daejeon, Korea). All chemicals and reagents used in this hepatic enzyme activity measurement were obtained from Sigma-Aldrich (St. Louise, MO, USA).

### Real-time RT-PCR (polymerase chain reaction) analysis

The ACC1, AMPKα1 and AMPKα2 mRNA expressions in the prepared hepatic tissues and the leptin, UCP2, adiponectin, C/EBPα, C/EBPβ and SREBP1c mRNA expressions in the periovarian adipose tissue were determined by *real-time* polymerase chain reaction (RT-PCR). Briefly, RNA was extracted using Trizol reagent (Invitrogen, Carlsbad, CA, USA). The RNA concentrations and quality were determined by CFX96^TM^ Real-Time System (Bio-Rad, Hercules, CA, USA). To remove contaminating DNA, samples were treated with recombinant DNase I (DNA-free; Ambion, Austin, TX, USA). RNA was reverse transcribed using the reagent High-Capacity cDNA Reverse Transcription Kit (Applied Biosystems, Foster City, CA, USA) according to the manufacturer’s instructions. The analysis was carried out using ABI Step One Plus Sequence Detection System (Applied Biosystems, Foster City, CA, USA), and their expression levels were calculated as relative to vehicle control. The following thermal conditions were applied: 10 min at 94°C and 39 cycles of 15 sec at 94°C, 20 sec at 57°C and 30 sec at 72°C. The data was normalized based on the GAPDH mRNA expression, using the comparative threshold cycle method. The oligonucleotide primer sequences used for PCR are listed in Table 1.

**Table 1.** Oligonucleotides used for Real-time RT-PCR in this study

Target	5’ – 3’	Sequence	GenBank Accession Number
Leptin	Sense	CCAAAACCCTCATCAAGACC	NM_008493
	Antisense	GTCCAACTGTTGAAGAATGTCCC	
UCP2	Sense	CCGCATTGGCCTCTACGACTCT	NM_011671
	Antisense	CCCCGAAGGCAGAAGTGAAGTG	
Adiponectin	Sense	CCCAAGGGAACTTGTGCAGGTTGGATG	NM_009605.4
	Antisense	GTTGGTATCATGGTAGAGAAGAAAGCC	
C/EBPα	Sense	TGGACAAGAACAGCAACGAGTAC	NM_001287523.1
	Antisense	CGGTCATTGTCACTGGTCAACT	
C/EBPβ	Sense	AAGCTGAGCGACGAGTACAAGA	NM_001287739.1
	Antisense	GTCAGCTCCAGCACCTTGTG	
SREBP1c	Sense	AGCCTGGCCATCTGTGAGAA	XM_006532714.2
	Antisense	CAGACTGGTACGGGCCACAA	
ACC1	Sense	GCCATTGGTATTGGGGCTTAC	NM_133360.2
	Antisense	CCCGACCAAGGACTTTGTTG	
AMPKα1	Sense	AAGCCGACCCAATGACATCA	XM_011245321.1
	Antisense	CTTCCTTCGTACACGCAAAT	
AMPKα2	Sense	GATGATGAGGTGGTGGA	NM_178143.2
	Antisense	GCCGAGGACAAAGTGC	
GAPDH	Sense	CATCTTCCAGGAGCGAGACC	NM_008084
	Antisense	TCCACCACCCTGTTGCTGTA	

RT-PCR = reverse transcription polymerase chain reaction

UCP = Mitochondrial uncoupling protein

C/EBP = CCAAT-enhancer-binding protein

SREBP = Sterol regulatory element-binding protein

ACC1 = Acetyl-CoA carboxylase 1

AMPK = 5' adenosine monophosphate-activated protein kinase

GAPDH = Glyceraldehyde 3-phosphate dehydrogenase

### Histopathology

After measuring of organ weights, some parts of the left lateral lobes of the liver, left kidney, splenic lobes of the pancreas, left periovarian fat pads and the abdominal wall deposited fat pads attached to the *muscularis quadratus lumborum* were fixed in 10% neutral buffered formalin. After paraffin embedding using automated tissue processor (Shandon Citadel 2000, Thermo Scientific, Waltham, MA, USA) and embedding center (Shandon Histocentre 3, Thermo Scientific, Waltham, MA, USA), 3-4 μm serial sections were prepared using a microtome (RM2255, Leica Biosystems, Nussloch, Germany). Representative sections were stained with hematoxylin and eosin (HE) for light microscopic examination (Eclipse 80i; Nikon, Tokyo, Japan). After that, the histological profiles of individual organs were observed. Alternatively, portions of the liver that had been dehydrated in 30% sucrose solutions were sectioned by cryostat for staining with oil red. To observe histopathological changes in more detail, the steatohepatitis regions and mean hepatocyte diameters (under HE staining) were calculated using an automated image analysis process (Model iSolution FL ver 9.1; IMT i-solution Inc., Vancouver, Quebec, 2012)^14,19,20^. Steatohepatitis regions, the percentage of fatty deposited regions in the hepatic parenchyma, were calculated as percentages of lipid deposited regions between the restricted histological view field of the liver (%/mm^2^ of hepatic parenchyma) under cryostat and oil red staining. The mean diameters of hepatocytes were also calculated in the restricted view fields on a computer monitor under paraffin embedding and HE staining using an automated image analysis process as μm; at least 10 hepatocytes per view field of the liver were considered. In addition, mean numbers of lipid droplets deposited in vacuolated renal tubules were also calculated using an automated image analysis process among 100 tubules (number/100 tubules; at 1 field of sample) and mean diameters of white adipocytes in each fat pad were calculated in the restricted view fields on a computer monitor using an automated image analysis process as μm; at least 10 white adipocytes per fat pad were considered. The thickness of deposited periovarian and abdominal wall fat pads (mm), the mean areas occupied by zymogen granules (%/mm2 of pancreatic parenchyma), the numbers of pancreatic islets (islets/10 mm2 of pancreatic parenchyma) and the diameters of pancreatic islets (μm) were also measured using an automated image analysis process. The histopathologist was blinded to group distribution when this analysis was made.

### Statistical analysis

All numerical values are expressed as mean ± standard deviation (SD) of 8 mice. Multiple comparison tests for different dose groups were conducted. Variance homogeneity was examined using the Levene test. If the Levene test indicated no significant deviations from variance homogeneity, the obtained data was analyzed by ANOVA test followed by least-significant differences multi-comparison (LSD) test to determine which comparison of pairs of groups was significantly different. In the case where significant deviations from variance homogeneity were observed using the Levene test, a non-parametric comparison test, Kruskal-Wallis H test, was conducted. When a significant difference was observed in the Kruskal-Wallis H test, the Mann-Whitney U (MW) test was conducted to determine the specific comparison of pairs of groups, which are significantly different. Statistical analyses were conducted using SPSS for Windows (Ver 22, IBM Corp, Armonk, NY, USA). Differences were considered significant at *p* < .05. 

## RESULTS

### Changes in organ and tissue weight

**Figure 2.**
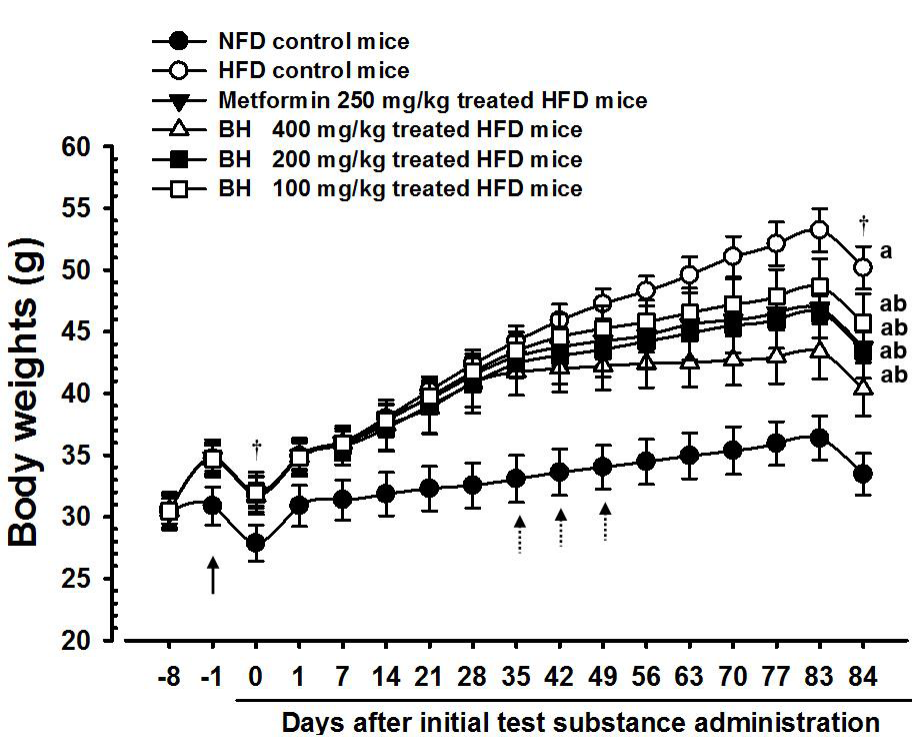
Body weight changes in NFD or HFD-fed mice NFD = Normal pellet diet; HFD = 45% kcal high fat diet BH = Domestic blue honeysuckle (Berries of Lonicera caerulea L.) extracts All animals were fasted overnight before initial test substance administrations and sacrifice (†) ^a^ p<0.01 as compared with the intact control group by LSD test ^b^ p<0.01 as compared with the HFD control group by LSD test

Adapted mice showed an increased regular body weight compared with healthy controls during the first week of HFD supply (healthy control: mean 30.88±1.55 g, range 28.70~33.10 g; HFD-fed group: mean 34.73±1.22 g, range 32.60~36.20 g); consequently, the HFD control mice showed a significant increase in body weight when compared with intact mice from the first week after HFD supply (*F=34.52, p<0.01*), and the body weight gains during 7 days of HFD adaption and 84 days of administration periods were also significantly increased compared with the intact control (*F=43.10, p <0.01*). However, significant decreases in body weights were detected in metformin 250 mg/kg-treated mice from 42 days after the start of administration, and from 35, 42 and 49 days after start of administration in BH extract 400, 200 and 100 mg/kg-treated mice when compared with the HFD control (*F=9.144, p<0.01 or F=6.654, p<0.05*). The body weight gain during 84 days of administration was also significantly decreased in the metformin 250 mg/kg and the BH extract 400, 200 and 100 mg/kg-treated mice compared with the HFD control (F=66.01, p<0.01). Especially, mice treated with all 3 different dosages of BH extract, 400, 200 and 100 mg/kg also showed obvious dose-dependent decreases in body weight and body weight gain during 84 days of the administration period. The metformin 250 mg/kg-treated group yielded comparable results to the BH 200 mg/kg-treated group in the present study (Table 2) (Fig. 2). The body weight gains during the 84 days of the administration period in the HFD control group were changed 229.08% when compared with the intact control group, but they were changed as -36.03, -53.03, -36.98 and -25.15% in the metformin 250 mg/kg-treated group and in the BH 400, 200 and 100 mg/kg-treated mice, respectively, when compared with the HFD control group. 

**Table 2.** Changes in body weight gain and mean daily food consumption in NFD or HFD-fed mice

Times	Body weight (g) at days after initial test substance treatment				Body weight gain during		Mean Daily Food Consumption (g)
Groups	8 days before [A]	1 day before [B]	0 day^*^ [C]	84 days^*^ [D]	Adaptive period [B-A]	Administration period [D-C]	
Controls							
Intact	30.48±1.49	30.88±1.55	27.86±1.48	33.45±1.69	0.45±0.20	5.59±0.83	5.58±0.29
HFD	30.46±1.03	34.75±1.04^a^	31.79±0.92^a^	50.18±1.73^a^	4.29±0.25^a^	18.39±1.48^a^	4.65±0.23^a^
Reference							
Metformin	30.40±1.35	34.78±1.26^a^	31.95±1.24^a^	43.71±1.25^a^^b^	4.38±0.20^a^	11.76±0.97^a^^b^	4.71±0.20^a^
Test material - BH							
400 mg/kg	30.49±1.49	34.70±1.38^a^	31.71±1.84^a^	40.35±2.18^a^^b^	4.21±0.17^a^	8.64±0.85^a^^b^	4.67±0.18^a^
200 mg/kg	30.45±1.50	34.74±1.82^a^	31.83±1.47^a^	43.41±2.20^a^^b^	4.29±0.17^a^	11.59±1.59^a^^b^	4.67±0.22^a^
100 mg/kg	30.45±1.31	34.68±1.19^a^	31.94±1.71^a^	45.70±2.37^a^^b^	4.23±0.31^a^	13.76±1.22^a^^b^	4.65±0.22^a^

Values are expressed as Mean ± SD of 8 mice

NFD = Normal pellet diet; HFD = 45% kcal high fat diet

BH extract = Domestic blue honeysuckle (Berries of Lonicera caerulea L.) extracts

Metformin was administered at a dose of 250 mg/kg

* All animals were fasted overnight

^a^ p<0.01 as compared with the intact control group at time-matched point by LSD test

^b^ p<0.01 as compared with the HFD control group at time-matched point by LSD test

The absolute liver weights also exhibited a significant increase in the HFD control mice compared with the healthy control mice (*F=543.82, p <0.05*). However, these increases in absolute liver weights were significantly normalized by treatment of all four test substances, including BH 400 mg/kg, compared with HFD control mice (*F=202.20, p<0.01*). Especially, mice treated with all three different dosages of BH 400, 200 and 100 mg/kg also showed definitive dose-dependent decreases in the liver absolute weights. The decreases in the metformin 250 mg/kg-treated group and the BH 200 mg/kg-treated group were comparable. No significant changes on the relative liver weights were noticed in mice treated with the 4 test materials when compared with those of HFD control mice (Table 2). The absolute liver weights in the HFD control changed by 66.49% compared with the intact control, but they were changed by -15.87, -23.08, -15.88 and -10.03% in the metformin 250 mg/kg-treated group and BH 400, 200 and 100 mg/kg-treated mice, respectively, compared with the HFD control group. The relative liver weights in the HFD control were changed by 10.84% compared with intact control, but they were changed by -3.54, -4.24, -2.52 and -1.05% in the metformin 250 mg/kg-treated group and the BH 400, 200 and 100 mg/kg-treated mice compared with the HFD control group. 

The periovarian and abdominal wall-stored fat pad relative and absolute weights in the HFD control mice also exhibited significant increases compared with the healthy control mice (*F=78.32, p<0.05*). However, these increases of the periovarian fat pad weight were significantly decreased by treatment of all test substances including treatment with BH extract 100 mg/kg, on both absolute and relative weights (*F=21.02, p<0.01*). Especially, mice treated with all three different BH extracts, 400, 200, and 100 mg/kg, also showed definitive dose-dependent decreases in the absolute and relative periovarian deposited fat pad weights. Such weight changes comparable to those observed in the metformin 250 mg/kg-treated group were also seen in the BH extract 200 mg/kg-treated group (Table 3). The absolute periovarian fat pad weight in the HFD control was changed by 678.22% compared with the intact control group, but they were changed by -43.18, -71.75, -42.45 and -23.46% in the metformin 250 mg/kg-treated group and the BH extract 400, 200 and 100 mg/kg-treated mice respectively compared with the HFD control group. The relative periovarian fat pad weights in the HFD control group were changed by 417.65% compared with the intact control group, but they were changed by -35.06, -64.94, -33.16 and -16.01% in the metformin 250 mg/kg-treated group and the BH extract 400, 200 and 100 mg/kg-treated mice respectively compared with the HFD control.

**Table 3.** Change on absolute and relative organ weights in NFD or HFD-fed mice

Organs	Absolute organ weights (g), Relative organ weights (% *of body weights*)					
Groups	Organ	Liver	Kidney	Pancreas	Periovarian fat pads	Abdominal wall fat pads
Controls						
Intact	Absolute	1.051±0.057	0.214±0.013	0.248±0.018	0.110±0.015	0.044±0.025
	Relative	3.151±0.251	0.641±0.029	0.745±0.079	0.329±0.053	0.130±0.067
HFD	Absolute	1.750±0.063^a^	0.326±0.012^a^	0.241±0.013	0.853±0.104^c^	0.705±0.147^c^
	Relative	3.492±0.181^a^	0.650±0.041	0.481±0.025^c^	1.703±0.221^c^	1.402±0.267^c^
Reference						
Metformin	Absolute	1.473±0.073^ab^	0.271±0.012^ab^	0.245±0.006	0.485±0.087^cd^	0.396±0.078^cd^
	Relative	3.369±0.141	0.620±0.034	0.560±0.017^cd^	1.106±0.173^cd^	0.906±0.182^cd^
Test material – BH						
400 mg/kg	Absolute	1.346±0.050^ab^	0.248±0.013^ab^	0.245±0.013	0.241±0.047^cd^	0.229±0.040^cd^
	Relative	3.344±0.210	0.615±0.039	0.607±0.035^cd^	0.597±0.111^cd^	0.569±0.098^cd^
200 mg/kg	Absolute	1.472±0.086^ab^	0.269±0.007^ab^	0.242±0.006	0.491±0.078^cd^	0.386±0.060^cd^
	Relative	3.404±0.336^b^	0.621±0.040	0.560±0.041^cd^	1.138±0.226^cd^	0.892±0.148^cd^
100 mg/kg	Absolute	1.575±0.079^ab^	0.292±0.016^ab^	0.244±0.014	0.653±0.043^cd^	0.494±0.064^cd^
	Relative	3.456±0.277^b^	0.640±0.056	0.533±0.024^cd^	1.431±0.092^cd^	1.081±0.131^cd^

Values are expressed as Mean ± SD of 8 mice

NFD = Normal pellet diet; HFD = 45% kcal high fat diet

BH extract = Domestic blue honeysuckle (Berries of Lonicera caerulea L.) extracts

Metformin was administrated at a dose level of 250 mg/kg

^a^ p<0.01 as compared with the intact control group at time-matched point by LSD test

^b^ p<0.01 as compared with the HFD control group at time-matched point by LSD test

^c^ p<0.01 as compared with the intact control group at time-matched point by MW test

^d^ p<0.01 as compared with the HFD control group at time-matched point by MW test

Similar changes to those observed in the periovarian deposited fat pads were observed in the abdominal wall deposited fat pad. Significant increases in abdominal wall deposited fat pad absolute and relative weights were detected in HFD controls compared with the intact control (*F=65.08, p<0.01*). However, these increases in the abdominal wall deposited fat pad absolute and relative weights were significantly decreased by treatment with all test substances, including metformin 250 mg/kg. (*F=11.08, p<0.01*). Especially, mice treated with all 3 different dosages of BH extract, 400, 200, and 100 mg/kg, also showed dose-dependent decreases in the absolute and relative abdominal wall deposited fat pad weights. The changes observed in metformin 250 mg/kg-treated group were comparable to those in the BH extract 200 mg/kg-treated group in the present experiment (Table 3). The absolute abdominal wall deposited fat pad weights in the HFD control were changed by 1492.94% compared with the intact control group, but they were changed by -43.87, -67.49, -45.22 and -29.93% in the metformin 250 mg/kg-treated group and the BH extract 400, 200 and 100 mg/kg-treated mice respectively compared with the HFD control group. The relative abdominal wall deposited fat pad weights in the HFD control group were changed by 974.72% compared with the intact control group but they were changed by -35.38, -59.43, -36.36 and -22.89% in the metformin 250 mg/kg-treated group and the BH extract 400, 200 and 100 mg/kg-treated mice, respectively, compared with the HFD control group.

### Effects on food consumption

Although significant decreases in the mean daily food consumptions were detected in all HFD-fed mice compared with the intact control group (*F=161.03, p<0.01*), no meaningful or significant changes on the mean daily food consumptions were detected in all test substance administered groups, including the BH extract 400 mg/kg treated group, when compared with the HFD control (Table 2). The mean daily food consumption during the 84 days of the administration period in the HFD control group was changed by -16.12% compared with the intact control group, but they were changed by 1.29, 0.31, 0.39 and -0.17% in the metformin 250 mg/kg-treated group and the BH extract 400, 200 and 100 mg/kg-treated mice respectively compared with the HFD control.

### Serum biochemical analysis

Significant increases in serum AST, ALT, ALP, LDH, GGT, and BUN levels were detected in the HFD control group (*F=10.94, p<0.01*). However, decreases in serum AST, ALT, ALP, LDH, GGT, BUN, and creatinine levels compared with the HFD control group were observed in all treatment groups. All BH extract treated groups resulted in a dose-dependent decrease in AST, ALT, ALP, LDH, GGT, BUN and creatinine levels compared with the HFD control mice (Table 4)

**Table 4.** Changes in Serum AST, ALT, ALP, LDH, GGT, BUN and Creatine levels in NFD or HFD-fed mice

Items	AST (IU/l)	ALT (IU/l)	ALP (IU/l)	LDH (IU/l)	GGT (IU/l)	BUN (mg/dl)	Creatinine (mg/dl)
Groups							
Controls							
Intact	58.63±14.69	33.50±11.64	88.63±20.37	298.25±125.20	1.75±0.71	33.13±10.36	0.64±0.30
HFD	185.13±25.77^a^	146.25±24.07^a^	219.38±31.95^a^	2139.50±376.26^a^	10.63±1.60^a^	99.38±15.90^a^	2.38±0.41^d^
Reference							
Metformin	128.63±20.63^a^^c^	99.00±15.45^a^^c^	150.75±26.08^a^^c^	1466.13±233.26^a^^c^	6.88±1.25^a^^c^	66.50±12.55^a^^c^	1.60±0.14^d^^e^
Test material – BH							
400 mg/kg	80.50±11.95^b^^c^	66.38±14.89^a^^c^	109.00±14.96^c^	990.88±159.80^a^^c^	4.75±1.39^a^^c^	47.13±10.80^b^^c^	1.14±0.22^d^^e^
200 mg/kg	127.00±14.67^a^^c^	98.75±10.94^a^^c^	145.75±23.85^a^^c^	1439.50±252.60^a^^c^	7.00±1.20^a^^c^	66.63±12.18^a^^c^	1.61±0.27^d^^e^
100 mg/kg	146.13±10.53^a^^c^	113.25±17.41^a^^c^	173.38±16.65^a^^c^	1652.50±228.50^a^^c^	7.88±1.36^a^^c^	76.13±9.31^a^^c^	1.88±0.16^d^^e^

Values are expressed as Mean ± SD of 8 mice

NFD = Normal pellet diet; HFD = 45% kcal high fat diet; ALT = Alanine aminotransferase; AST = Aspartate aminotransferase; ALP = Alkaline phosphatase; LDH = Lactate dehydrogenase; GGT = Gamma-glutamyltransferase; BUN = Blood urea nitrogen

BH extract = Domestic blue honeysuckle (Berries of Lonicera caerulea L.) extracts

Metformin was administrated at a dose level of 250 mg/kg

^a^ p<0.01 and ^b^ p<0.05 as compared with the intact control group at time-matched point by LSD test

^c^ p<0.01 as compared with the HFD control group at time-matched point by LSD test

^d^ p<0.01 as compared with the intact control group at time-matched point by MW test

^e^ p<0.01 as compared with the HFD control group at time-matched point by MW test

### Serum biochemical analysis

Significant increases in serum total cholesterol (TC), triglycerides (TG), low-density lipoprotein (LDL) levels were detected in the HFD control group (*F=13.02, p<0.01*). However, a decrease in serum TC, TG, and LDL-C levels compared with the HFD control group were observed in all treatment groups (Table 5). Although non-significant slight increases of fecal TC and TG contents were detected in the HFD control group compared with the intact control group, the fecal TC and TG contents in mice treated with all 4 test materials, including metformin 250 mg/kg, were significantly elevated compared with the HFD control mice (*F=7.67, p<0.01 or F=7.26, p<0.05*). Especially, all three different groups of mice treated with BH extracts, 400, 200 and 100 mg/kg, also showed obvious dose-dependent increases in the fecal TC and TG contents. The increase in the metformin 250 mg/kg treated group was comparable to that of the BH extract 200 mg/kg-treated group. The fecal TC contents in the HFD control were changed by 13.57% as compared with the intact control group, but they were changed by 110.19, 245.38, 119.27 and 54.55% in the metformin 250 mg/kg-treated group and the BH extract 400, 200 and 100 mg/kg-treated mice, respectively, compared with the HFD control group. The fecal TG contents in HFD control were changed by 14.00% compared with the intact control group, but they were changed by 107.00, 247.95, 108.08 and 55.28% in the metformin 250 mg/kg-treated group and the BH extract 400, 200 and 100 mg/kg-treated mice, respectively, compared with the HFD control group.

**Table 5.** Changes in blood glucose levels and serum lipid contents in NFD or HFD-fed mice

Items	Glucose (mg/dl)	Total cholesterol (mg/dl)	Triglyceride (mg/dl)	Low density lipoprotein (mg/dl)	High density lipoprotein (mg/dl)
Groups					
Controls					
Intact	91.75±16.24	97.25±14.15	72.59014.96	17.63±3.81	89.25±18.87
HFD	268.38±35.62^e^	263.00±33.09^e^	245.75±27.69^a^	79.38±10.20^a^	16.75±2.96^a^
Reference					
Metformin	179.63±21.31^e^^f^	178.13±19.66^e^^f^	166.75±29.51^a^^c^	53.88±12.09^a^^c^	41.25±14.20^a^^c^
Test material - BH					
400 mg/kg	128.25±10.78^e^^f^	123.88±13.25^e^^f^	117.75±20.32^a^^c^	38.00±11.43^a^^c^	70.63±17.42^b^^c^
200 mg/kg	177.25±26.69^e^^f^	179.88±26.82^e^^f^	164.00±21.11^a^^c^	52.13±11.47^a^^c^	41.38±12.37^a^^c^
100 mg/kg	210.38±37.28^e^^g^	209.38±19.92^e^^f^	194.88±12.57^a^^c^	60.50±13.86^a^^c^	30.38±10.20^a^^d^

Values are expressed as Mean ± SD of 8 mice

NFD = Normal pellet diet; HFD = 45% kcal high fat diet

BH = Domestic blue honeysuckle (Berries of Lonicera caerulea L.) extracts

Metformin was administrated at a dose level of 250 mg/kg

^a^ p<0.01 and ^b^ p<0.05 as compared with the intact control group at time-matched point by LSD test

^c^ p<0.01 and ^d^ p<0.05 as compared with the HFD control group at time-matched point by LSD test

^e^ p<0.01 as compared with the intact control group at time-matched point by MW test

^f^ p<0.01 and ^g^ p<0.05 as compared with the HFD control group at time-matched point by MW test

### Effects on lipid peroxidation and the antioxidant defense system

Significant increases in liver lipid peroxidation, seen as hepatic MDA content elevations, were detected in the HFD control group compared with the intact control group (*F=18.88, p<0.01*), but they were significantly normalized by treatment of mice with all four test materials, including the BH extract 400 mg/kg, when compared with the HFD control mice group (*F=17.51, p<0.01*). Especially, mice treated with all 3 different dosages of BH extracts, 400, 200 and 100 mg/kg, also showed obvious dose-dependent decreases in hepatic lipid peroxidation, seen as hepatic MDA contents, where those in the metformin 250 mg/kg treated group yielded comparable results to those in the BH extract 200 mg/kg-treated group (Table 6). The hepatic lipid peroxidation in the HFD control group was changed by 1153.81% compared with the intact control, but they were changed by -33.17, -58.62, -34.45 and -23.23% in the metformin 250 mg/kg and BH extract 400, 200 and 100 mg/kg-treated mice respectively compared with the HFD control group. 

**Table 6.** Changes in the liver lipid peroxidation and antioxidant defense systems in NFD or HFD-fed mice

Items	Lipid peroxidation	Antioxidant defense system		
Groups	Malondialdehyde (nM/mg tissue)	Glutathione (μM/mg tissue)	Catalase (U/mg tissue)	SOD (U/mg tissue)
Controls				
Intact	6.08±1.91	69.95±13.05	67.48±18.37	7.57±1.05
HFD	76.25±10.33^d^	11.48±3.76^a^	11.34±2.97^d^	1.24±0.47^a^
Reference				
Metformin	50.95±10.97^d^^e^	30.19±10.65^a^^b^	31.51±13.58^d^^e^	3.39±0.97^a^^b^
Test material - BH				
400 mg/kg	31.55±10.98^d^^e^	50.39±10.79^a^^b^	48.15±12.16^e^	5.03±1.37^a^^b^
200 mg/kg	49.98±12.40^d^^e^	31.77±11.17^a^^b^	31.34±8.02^d^^e^	3.43±0.65^a^^b^
100 mg/kg	58.53±12.66^d^^e^	24.56±9.04^a^^c^	23.36±8.84^d^^e^	2.57±0.97^a^^b^

Values are expressed as Mean ± SD of 8 mice

NFD = Normal pellet diet; HFD = 45% kcal high fat diet; SOD = Superoxide dismutase

BH extract = Domestic blue honeysuckle (Berries of Lonicera caerulea L.) extracts

Metformin was administrated at a dose level of 250 mg/kg

^a^ p<0.01 as compared with the intact control group at time-matched point by LSD test

^b^ p<0.01 and ^c^ p<0.05 as compared with the HFD control group at time-matched point by LSD test

^e^ p<0.01 as compared with the HFD control group at time-matched point by MW test

^d^ p<0.01 as compared with the intact control group at time-matched point by MW test

Significant decreases in hepatic GSH contents, a representative endogenous antioxidant, were detected in the HFD control group compared with the intact control group (*F=7.29, p<0.01*). However, the hepatic GSH contents were significantly and dramatically increased in mice treated with all test substances, including the BH extract 200 mg/kg, when compared with the HFD control mice (*F=11.01, p<0.01 or F=8.81, p<0.05*). Especially, all 3 different groups of mice treated with BH extract, 400, 200 and 100 mg/kg, also showed definitive dose-dependent increases of the hepatic GSH contents. The metformin 250 mg/kg treated group yielded comparable results to the BH extract 200 mg/kg-treated group (Table 6). The hepatic GSH contents in the HFD control group were changed by -83.59% compared with the intact control group, but they were changed by 163.01, 339.01, 176.80 and 113.99% in the metformin 250 mg/kg and BH extract 400, 200 and 100 mg/kg-treated mice, respectively, when compared with the HFD control group. 

Significant decreases in hepatic CAT activities, a representative endogenous antioxidant enzyme, were detected in the HFD control group compared with the intact control group (*F=9.10, p<0.01*), but these decreases in hepatic CAT activities were significantly normalized by treatment with all four test materials, including the BH extract 100 mg/kg, when compared with the HFD control group (*F=10.02, p<0.01*). Especially, mice treated with all three different dosages of BH extract, 400, 200 and 100 mg/kg, also showed dose-dependent increases in hepatic CAT activities. The metformin 250 mg/kg-treated group yielded similar results to the BH extract 200 mg/kg-treated group (Table 6). The hepatic CAT activities in the HFD control group were changed by -83.20% compared with the intact control group, but they were changed by 177.96, 324.77, 176.45 and 106.08% in the metformin 250 mg/kg and BH 400, 200 and 100 mg/kg-treated mice, respectively, compared with the HFD control group. 

Significant decreases in hepatic SOD activities, another representative endogenous antioxidant enzyme, were detected in the HFD control group compared with the intact control group (*F=12.71, p<0.01*), but they were significantly normalized by treatment with all four test materials, including metformin 250 mg/kg, when compared with the HFD control mice (*F=9.10, p<0.01*). Especially, mice treated with all 3 different dosages of BH extract, 400, 200 and 100 mg/kg, also showed clear dose-dependent increases in the hepatic SOD activities. The metformin 250 mg/kg-treated group yielded comparable results to the BH extract 200 mg/kg-treated group (Table 6). The hepatic SOD activities in the HFD control group were changed by -83.63% as compared with the intact control, but they were changed by 173.59, 305.85, 176.53 and 107.46% in the metformin 250 mg/kg and BH extract 400, 200 and 100 mg/kg-treated mice, respectively, compared with the HFD control group.

### Effects on body fat density and total and abdominal fat mass

Significant increases in total body and abdominal fat densities were detected in the HFD control group as compared with the intact control group (*F=8.10, p<0.01*). On the contrary, a significant decrease of total body and abdominal fat masses were detected in mice treated with all test substances, including BH extract 200 mg/kg, by analysis of in live DEXA (*F=8.70, p<0.0*1 or *F=7.24, p<0.05*). Especially, all 3 different groups of mice treated with BH extract, 400, 200 and 100 mg/kg, also showed clear dose-dependent decreases in total body and abdominal fat masses. The metformin 250 mg/kg-treated group yielded comparable results to the BH extract 200 mg/kg-treated group. The mean total body fat densities of the HFD control group were changed by 232.45% compared with the intact control group, but they were changed by -39.00, -56.56, -38.18 and -24.05% in the metformin 250 mg/kg and BH extract 400, 200 and 100 mg/kg-treated mice, respectively, compared with the HFD control group. The mean abdominal fat densities of the HFD control group were changed by 282.64% compared with the intact control group, but they were changed by -41.39, -57.12, -43.06 and -23.16% in the metformin 250 mg/kg and BH extract 400, 200 and 100 mg/kg-treated mice, respectively, compared with the HFD control group. 

### Effects on adipocyte histopathology analysis

Significant increases in periovarian and abdominal white adipocyte diameters and thicknesses of each deposited fat pads were detected in the HFD control group compared with the intact control group (*F=9.29, p<0.01*). However, this hypertrophy of adipocytes and fat depositions were significantly inhibited by treatment with all 4 test substances including BH extract 400 mg/kg, compared with the HFD control group (*F=10.12, p<0.01*). Especially, all 3 different groups of mice treated with the BH extract, 400, 200 and 100 mg/kg, also showed clear dose-dependent decreases in the periovarian and abdominal wall deposited white adipocyte diameters and thicknesses of each deposited fat pad. The metformin 250 mg/kg-treated group yielded comparable results to the BH 200 mg/kg-treated group (Table 7). The deposited periovarian fat pad thickness in the HFD control group was changed by 256.05% compared with the intact control group, but was changed by -30.74, -42.00, -31.12 and -22.59% in the metformin 250 mg/kg and BH extract 400, 200 and 100 mg/kg-treated mice, respectively, compared with the HFD control group. The mean periovarian white adipocyte diameter in the HFD control group was changed by 206.39% compared with the intact control group, but was changed by -40.93, -52.06, -42.03 and -27.77% in the metformin 250 mg/kg and BH extract 400, 200 and 100 mg/kg-treated mice, respectively, compared with the HFD control group. The abdominal wall deposited fat pad thickness in the HFD control group was changed by 267.34% compared with the intact control group, but was changed by -23.67, -35.23, -23.53 and -19.68% in the metformin 250 mg/kg and BH extract 400, 200 and 100 mg/kg-treated mice, respectively, compared with the HFD control group. The mean abdominal wall deposited fat pad white adipocyte diameter in the HFD control group was changed by 204.72% compared with the intact control group, but was changed by -30.68, -45.09, -32.85 and -19.82% in the metformin 250 mg/kg and BH extract 400, 200 and 100 mg/kg-treated mice, respectively, compared with the HFD control group.

**Table 7.** Changes in the histopathology-histomorphometry of the periovarian and abdominal wall deposited fat pads in NFD or HFD-fed mice

Items	Lipid peroxidation	Antioxidant defense system		
Groups	Thickness (mm)	Adipocyte diameters (μm)	Thickness (mm)	Adipocyte diameters (μm)
Controls				
Intact	1.27±0.48	33.23±5.80	1.61±0.77	36.12±3.86
HFD	4.53±0.77^a^	101.82±13.22^c^	5.92±0.60^a^	110.08±9.07^a^
Reference				
Metformin	3.14±0.64^a^^b^	60.14±7.07^c^^d^	4.52±0.56^a^^b^	76.30±16.79^a^^b^
Test material - BH				
400 mg/kg	2.63±0.47^a^^b^	48.82±7.52^c^^d^	3.83±0.73^a^^b^	60.45±11.41^a^^b^
200 mg/kg	3.12±0.47^a^^b^	59.03±7.35^c^^d^	4.53±0.51^a^^b^	73.91±8.46^a^^b^
100 mg/kg	3.50±0.56^a^^b^	73.55±4.10^c^^d^	4.75±0.79^a^^b^	88.26±8.06^a^^b^

Values are expressed as Mean ± SD of 8 mice

NFD = Normal pellet diet; HFD = 45% kcal high fat diet

BH extract = Domestic blue honeysuckle (Berries of Lonicera caerulea L.) extracts

Metformin was administrated at a dose level of 250 mg/kg

^a^ p<0.01 as compared with the intact control group at time-matched point by LSD test

^b^ p<0.01 as compared with the HFD control group at time-matched point by LSD test

^c^ p<0.01 as compared with the intact control group at time-matched point by MW test

^d^ p<0.01 as compared with the HFD control group at time-matched point by MW test

### Effects on the exocrine pancreas zymogen granule content

Significant decreases in the exocrine pancreas zymogen granule contents (the percentages of exocrine pancreas occupied by zymogen granules) were detected in the HFD control group compared with the intact control group, resulting from a release of zymogen granules (*F=12.07, p<0.01*). However, exocrine pancreas zymogen granule contents were significantly increased in all test drug-treated mice groups compared with the HFD control group, including the BH extract 200 mg/kg treated group (*F=11.04, p<0.01*). Especially, all three different groups of BH extract 400, 200 and 100 mg/kg-treated mice also showed obvious dose-dependent increases in the percentage of the regions of exocrine pancreas occupied by zymogen granules. The metformin 250 mg/kg-treated group yielded comparable results to the BH extract 200 mg/kg treated mice, in our experiment (Table 8). The percentage regions of exocrine pancreas occupied by zymogen granule in the HFD control group were changed by -63.71% compared with the intact control group, but they were changed by 83.41, 135.80, 86.31 and 58.61% in the metformin 250 mg/kg and BH 400, 200 and 100 mg/kg-treated mice, respectively, compared with the HFD control group. 

**Table 8.** Changes in histopathology-histomorphometry of the pancreas in NFD or HFD-fed mice

Items	Zymogen granules (%/mm^2^ of exocrine)	Mean islet numbers (numbers/10 mm^2^)	Mean islet diameter (μm/islet)	Insulin-IR cells (cells/ mm^2^) [A]	Glucagon-IR cells (cells/ mm^2^) [B]	Insulin/glucagon ratio [A/B]
Groups						
Controls						
Intact	45.75±4.23	11.13±2.53	96.70±15.38	82.38±15.57	24.13±5.11	3.44±0.24
HFD	16.61±5.36^a^	29.25±3.54^a^	319.59±50.14^a^	923.38±112.49^d^	141.50±8.82^a^	6.51±0.46^a^
Reference						
Metformin	30.46±4.78^a^^c^	21.88±3.87^a^^c^	216.11±30.10^a^^c^	392.13±68.17^d^^e^	82.00±12.41^a^^c^	4.79±0.54^a^^c^
Test material - BH						
400 mg/kg	39.16±5.40^a^^c^	17.50±2.62^a^^c^	158.47±26.39^a^^c^	180.63±33.33^d^^e^	45.75±9.71^a^^c^	3.99±0.40^b^^c^
200 mg/kg	30.94±3.27^a^^c^	21.63±2.00^a^^c^	212.73±38.47^a^^c^	412.13±39.41^d^^e^	86.88±3.94^a^^c^	4.75±0.41^a^^c^
100 mg/kg	26.34±4.46^a^^c^	24.13±2.17^a^^c^	244.31±34.20^a^^c^	653.00±94.68^d^^e^	122.63±10.68^a^^c^	5.30±0.36^a^^c^

Values are expressed as Mean ± SD of 8 mice

NFD = Normal pellet diet; HFD = 45% kcal high fat diet

BH extract = Domestic blue honeysuckle (Berries of Lonicera caerulea L.) extracts

Metformin was administrated at a dose level of 250 mg/kg

^a^ p<0.01 and ^b^ p<0.05 as compared with the intact control group at time-matched point by LSD test

^c^ p<0.01 as compared with the HFD control group at time-matched point by LSD test

^d^ p<0.01 as compared with the intact control group at time-matched point by MW test

^e^ p<0.01 and ^f^ p<0.05 as compared with the HFD control group at time-matched point by MW test

### Effects on hepatocyte hypertrophy

**Figure 3.**
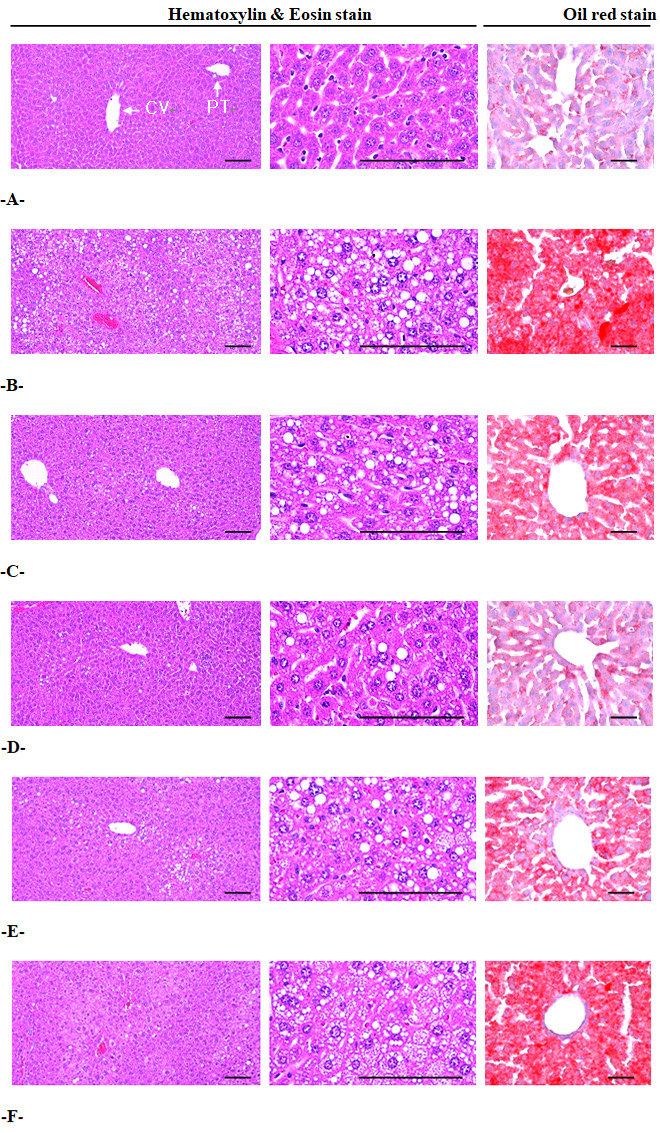
A = Intact control: 10 mL/kg of distilled water orally administered to mice with NFD supply B = HFD control: 10 mL/kg of distilled water orally administered to mice with HFD supply C = Metformin: 250 mg/kg of metformin orally administered to mice with HFD supply D = BHd400: 400 mg/kg of BH orally administered to mice with HFD supply E = BHd200: 200 mg/kg of BH orally administered to mice with HFD supply F = BHd100: 100 mg/kg of BH orally administered to mice with HFD supply NFD = Normal pellet diet; HFD = 45% kcal high fat diet; CV = Central vein; PT = Portal triad BH = Domestic blue honeysuckle (Berries of *Lonicera caerulea L*.) extracts Scale bars = 100 µm.

Significant increases in mean hepatocyte diameters (hypertrophy) were detected in the HFD control group compared with the intact control group (*F=10.11, p<0.01*). However, these hepatocyte hypertrophies were markedly and significantly decreased in mice treated with all 4 test substances, including metformin 250 mg/kg, compared with the HFD control group (*F=8.87, p<0.01*). Especially, mice treated with all three different dosages of the BH extract, 400, 200 and 100 mg/kg, also showed clear dose-dependent decreases in hepatocyte hypertrophy, the mean diameters of hepatocytes. The metformin 250 mg/kg-treated group yielded comparable results to the BH extract 200 mg/kg-treated group (Table 9) (Fig. 3). The mean hepatocyte diameter in the HFD control group was changed by 93.60% compared with the intact control group, but was changed by -22.14, -29.73, -22.48 and -15.47% in metformin 250 mg/kg and BH extract 400, 200 and 100 mg/kg-treated mice, respectively, compared with the HFD control group.

**Table 9.** Changes in histopathology-histomorphometry of the liver and kidney in NFD or HFD-fed mice

Items	Liver steatosis (%/mm2 of hepatic tissues)	Mean hepatocyte diameters (μm/cell)	Degenerative renal tubule numbers (%)
Groups			
Controls			
Intact	9.80±4.57	17.33±2.11	4.50±2.20
HFD	80.05±10.65^a^	33.56±2.05^a^	73.50±11.84^a^
Reference			
Metformin	53.66±13.38^a^^b^	26.13±2.62^a^^b^	48.38±11.25^a^^b^
Test material - BH			
400 mg/kg	34.57±10.38^a^^b^	23.58±2.13^a^^b^	30.00±11.11^a^^b^
200 mg/kg	53.94±10.77^a^^b^	26.02±1.44^a^^b^	48.13±14.47^a^^b^
100 mg/kg	62.86±10.58^a^^b^	28.37±1.98^a^^b^	57.00±10.34^a^^b^

Values are expressed as Mean ± SD of 8 mice

NFD = Normal pellet diet; HFD = 45% kcal high fat diet

BH extract = Domestic blue honeysuckle (Berries of Lonicera caerulea L.) extracts

Metformin was administrated at a dose level of 250 mg/kg

^a^ p<0.01 as compared with the intact control group at time-matched point by LSD test

^b^ p<0.01 as compared with the HFD control group at time-matched point by LSD test

Significant increases in steatohepatitis (percentages of fatty changed regions in the liver parenchyma) were detected in the HFD control group compared with the intact control group, resulting from severe hypertrophy of hepatocytes related to the intracellular lipid depositions (*F=12.87, p<0.01*). However, the presence of steatohepatitis was significantly normalized by treatment with all 4 test substances, including BH extract 100 mg/kg, compared with the HFD control group (*F=11.82, p<0.01*). Especially, all three different groups of BH extract 400, 200 and 100 mg/kg-treated mice also showed dose-dependent decreases in the steatohepatitis regions, where the metformin 250 mg/kg-treated group yielded comparable results to the BH extract 200 mg/kg-treated group (Table 9, Fig. 3). The steatohepatitis regions in the HFD control group were changed by 716.49% compared with the intact control group, but they were changed by -32.96, -56.81, -32.61 and -21.48% in the metformin 250 mg/kg and BH extract 400, 200 and 100 mg/kg-treated mice, respectively, compared with the HFD control group.

### Effects on hepatic enzyme activity

Significant decreases in hepatic Glucokinase (GK) activities, a hepatic enzyme utilizing blood glucose, were detected in the HFD control group compared with the intact control group (*F=13.01, p<0.01*), but they were significantly normalized by treatment with all 4 test materials, including BH extract 400 mg/kg, compared with the HFD control mice (*F=10.88, p<0.01* or *F=6.95, p<0.05*). Especially, mice treated with all 3 different dosages of BH extract, 400, 200 and 100 mg/kg, also showed obvious dose-dependent increases in hepatic GK activities. The metformin 250 mg/kg-treated group yielded comparable results to the BH extract 200 mg/kg treated group (Table 10). The hepatic GK activities in the HFD control group were changed by -84.03% compared with the intact control, but they were changed by 121.17, 226.63, 120.11 and 66.58% in the metformin 250 mg/kg and BH extract 400, 200 and 100 mg/kg treated mice, respectively, compared with the HFD control group.

**Table 10.** Changes in the hepatic glucose-regulating enzyme activities in NFD or HFD-fed mice

Items	Glucokinase (nM/min/mg protein)	Glucose-6-phosphatase (nM/min/mg protein)	PEPCK (nM/min/mg protein)
Groups			
Controls			
Intact	5.88±1.63	121.38±31.97	2.08±1.15
HFD	0.94±0.48^d^	387.30±83.00^d^	7.98±1.21^a^
Reference			
Metformin	2.08±0.49^d^^f^	211.27±29.00^d^^f^	4.63±1.26^a^^c^
Test material - BH			
400 mg/kg	3.07±0.65^d^^f^	158.96±25.68^e^^f^	3.35±0.54^b^^c^
200 mg/kg	2.07±0.55^d^^f^	210.00±40.65^d^^f^	4.46±0.60^a^^c^
100 mg/kg	1.56±0.26^d^^g^	256.57±38.10^d^^f^	5.21±0.96^a^^c^

Values are expressed as Mean ± SD of 8 mice

NFD = Normal pellet diet; HFD = 45% kcal high fat diet; PEPCK = Phosphoenolpyruvate carboxykinase

BH extract = Domestic blue honeysuckle (Berries of Lonicera caerulea L.) extracts

Metformin was administrated at a dose level of 250 mg/kg

^a^ p<0.01 and ^b^ p<0.05 as compared with the intact control group at time-matched point by LSD test

^c^ p<0.01 as compared with the HFD control group at time-matched point by LSD test

^d^ p<0.01 and ^e^ p<0.05 as compared with the intact control group at time-matched point by MW test

^f^ p<0.01 and ^g^ p<0.05 as compared with the HFD control group at time-matched point by MW test

Significant increases in hepatic G6pase activities, one of the gluconeogenesis hepatic enzymes, were detected in the HFD control group compared with the intact control group (*F=9.64, p<0.01*), but they were significantly normalized by treatment with all four test materials, including BH extract 200 mg/kg, compared with the HFD control mice (F*=8.98, p<0.01*). Especially, mice treated with all 3 different dosages of BH extract, 400, 200 and 100 mg/kg, also showed obvious dose-dependent decreases in the hepatic G6pase activities. The metformin 250 mg/kg-treated group yielded comparable results to the BH extract 200 mg/kg treated group, in this detection (Table 10). The hepatic G6pase activities in the HFD control group were changed by 219.07% compared with the intact control, but they were changed by -45.45, -58.96, -45.78 and -33.76% in the metformin 250 mg/kg and BH extract 400, 200 and 100 mg/kg-treated mice respectively compared with the HFD control group. Significant increases in hepatic PEPCK activities, another gluconeogenesis hepatic enzyme, were detected in the HFD control group compared with the intact control group (*F=11.18, p<0.01*), but they were significantly normalized by treatment with all four test materials, including BH extract 100 mg/kg, compared with the HFD control mice (*F=10.49, p<0.01*). Especially, mice treated with all three different groups of BH extract, 400, 200 and 100 mg/kg, also showed obvious dose-dependent decreases in the hepatic PEPCK activities. The metformin 250 mg/kg-treated group yielded comparable results to the BH extract 200 mg/kg treated group (Table 10). The hepatic PEPCK activities in the HFD control group were changed by 284.00% compared with the intact control, but they were changed by -42.05, -58.02, -44.11 and -34.78% in the metformin 250 mg/kg and BH extract 400, 200 and 100 mg/kg-treated mice, respectively, compared with the HFD control group.

### Effects on expression of lipid metabolism-associated gens

Significant increases in hepatic tissue ACC1, adipose tissue leptin, C/EBPα, C/EBPβ (*F=12.11, p<0.01*), and SREBP1 mRNA expressions and a significant decrease in hepatic AMPKα1, AMPKα2, adipose tissue UCP2, and adiponectin mRNA expressions were observed in the HFD control group (*F=7.21, p<0.05*) (Table 11). Specifically, all 3 different groups of BH extract 400, 200 and 100 mg/kg-treated mice also showed dose-dependent decreases in the mRNA expression of hepatic tissue ACC1, adipose tissue leptin, C/EBPα, C/EBPβ, and SREBP1 and dose-dependent increases in the mRNA expression of hepatic AMPKα1, AMPKα2, adipose tissue UCP2, and adiponectin compared with the metformin (250 mg/kg)-treated group using RT-qPCR analysis (Table 11).

**Table 11.** Changes in lipid metabolism-related gene mRNA expressions in NFD or HFD-fed mice, Real-time RT-PCR analysis

Items	Controls		Reference	Test material - BH		
Groups	Intact	HFD	Metformin	400 mg/kg	200 mg/kg	100 mg/kg
Hepatic tissue						
ACC1	1.00±0.08	4.64±0.94^e^	2.93±0.53^e^^f^	1.98±0.55^e^^f^	2.92±0.62^e^^f^	3.53±0.44^e^^f^
AMPKα1	1.06±0.09	0.25±0.08^a^	0.47±0.09^a^^c^	0.75±0.17^a^^c^	0.47±0.12^a^^c^	0.40±0.10^a^^d^
AMPKα2	0.99±0.06	0.34±0.11^a^	0.63±0.12^a^^c^	0.82±0.15^a^^c^	0.63±0.11^a^^c^	0.53±0.07^a^^c^
Adipose tissue						
Leptin	1.00±0.07	6.70±0.99^e^	4.37±0.59^e^^f^	2.69±0.96^e^^f^	4.23±0.79^e^^f^	5.04±0.90^e^^f^
UCP2	1.00±0.06	0.13±0.05^a^	0.36±0.10^a^^c^	0.54±0.16^a^^c^	0.35±0.08^a^^c^	0.24±0.04^a^^d^
Adiponectin	0.98±0.08	0.14±0.04^a^	0.40±0.08^a^^c^	0.60±0.10^a^^c^	0.41±0.12^a^^c^	0.26±0.07^a^^c^
C/EBPα	1.03±0.10	3.28±0.82^a^	2.05±0.36^a^^c^	1.46±0.20^b^^c^	2.04±0.20^a^^c^	2.34±0.28^a^^c^
C/EBPβ	1.03±0.07	4.28±1.00^e^	2.74±0.54^e^^f^	2.00±0.43^e^^f^	2.77±0.52^e^^f^	3.12±0.24^e^^f^
SREBP1c	1.02±0.09	3.79±0.76^e^	2.39±0.43^e^^f^	1.81±0.33^e^^f^	2.42±0.43^e^^f^	2.75±0.50^e^^f^

Values are expressed as Mean ± SD of 8 mice

NFD = Normal pellet diet; HFD = 45% kcal high fat diet; RT-PCR = reverse transcription polymerase chain reaction; UCP = Mitochondrial uncoupling protein; C/EBP = CCAAT-enhancer-binding protein; SREBP = Sterol regulatory element-binding protein; ACC1 = Acetyl-CoA carboxylase 1; AMPK = 5' adenosine monophosphate-activated protein kinase; GAPDH = Glyceraldehyde 3-phosphate dehydrogenase

BH extract = Domestic blue honeysuckle (Berries of Lonicera caerulea L.) extracts

Metformin was administrated at a dose level of 250 mg/kg

^a^ p<0.01 and ^b^ p<0.05 as compared with the intact control group at time-matched point by LSD test

^c^ p<0.01 and ^d^ p<0.05 as compared with the HFD control group at time-matched point by LSD test

^e^ p<0.01 as compared with the intact control group at time-matched point by MW test

^f^ p<0.01 as compared with the HFD control group at time-matched point by MW test

## DISCUSSION

In this study, we used a high-fat diet mouse model to determine the effects of BH extract intake on hepatic lipid metabolism. Results confirmed improved mRNA levels of ACC1, AMPK α1, AMPK α2, adipose tissue Leptin, UCP2, Adiponectin, C / EBP α, C / EBPβ, and SREBP1c. 

The major cause of NAFLD is known to be obesity^21^, and efforts to reduce type 2 diabetes and fat mass for the prevention and treatment of NAFLD have been recommended^22,23^. Our results showed that BH extract intake significantly lowers serum insulin levels and fat mass and that BH extract 200 mg/kg-treated mice had similar insulin levels and had inhibited increased fat weight, similar to the metformin 250 mg/kg group. BH extract has been identified as a potential candidate for NAFLD prevention and treatment. The most bioactive component (predominant anthocyanin) of BH extract is cyanidin-3-glucoside (C3G)^24,25^. C3G ingestion is known to improve obesity and triglyceride metabolism by increasing phosphorylation of adenosine monophosphate protein kinase in skeletal muscle and visceral adipose tissue^26^. In addition, in an in vitro study^27^, C3G was also shown to reduce the amount of reactive oxygen species produced in adipocytes, suggesting that it affected serum insulin levels and fat mass changes in this study. 

Although our results did not confirm the direct effects on Nrf2 expression and nuclear translocation upon increased AMPK activity by BH extract ingestion in this study, we found that, using a reporter gene assay for BH extract-dependent Nrf2 expression, in our recent in vitro study^12^ the expression of the reporter gene (luciferase activity) was increased in a dose-dependent manner. Therefore, we hypothesized that the contribution of mediating activation of AMPK by BH extract improves NAFLD through bettering hepatic lipid metabolism. Our expectation was due to the strong antioxidant response due to high vitamin C content, total phenolic content, and total anthocyanin in BH^7^. In particular, NAFLD is characterized by the accumulation of TG in the liver^2^. In this study, BH extract showed a dose-dependent effect of decreasing total cholesterol and LDL-cholesterol as well as TG, as well as increasing HDL-cholesterol, suggesting a fundamental improvement of NAFLD. In our study, we could not confirm the activation of lipoprotein lipase in adipocytes by BH extract ingestion. However, increased expression of AMPK α1 and AMPK α2 in hepatic tissue after BH extract ingestion showed the possibility of lipoprotein suppression in adipocytes. Therefore, changes in cholesterol may be considered to have occurred. Although not the same BH extract was used in this study, the NAFLD improvement effect of Kim and Colleagues^10^ using a similar BH extract also showed a dose-dependent decrease in TG, total cholesterol, and LDL-cholesterol and an increase in HDL-cholesterol. In addition, changes in gene expression related to lipid metabolism from adipose tissue were also confirmed, suggesting that the potential for hepatic lipid metabolism activity is clear. Wu and colleagues^9^ compared the study of Kim et al.^10^ with a high-fat diet as well as a combination of carbon tetrachloride (CCL4) to induce nonalcoholic steatohepatitis (NASH). As a result, they reported that the effect of TG reduction according to BH extract complex intake was the same. Based on these findings, it would be clear that the improvement of NAFLD is due to the reduction of lipid metabolism through BH extract injection. 

We have confirmed the down-regulation of lipid peroxidation and inflammation by down-regulation of FoxO1 and HO-1 in NASH due to Nrf2 up-regulation by polyphenols from BH as the mechanism of NAFLD improvement by ingestion of BH extract. Based on these results, we accepted the previous study's claim that ingestion of BH extract was effective in improving NASH^9^. We studied the changes in Nrf2 up-regulation and antioxidant enzyme expression using Korean BH and Chinese BH through in vitro studies undertaken before this study. As a result, Korean BH was shown to be superior to Chinese BH^12^. Nrf2 behaves identically to the increased antioxidant enzyme expression. In our study and in that of Kim et al.^10^, lipid peroxidation decreased after BH extract ingestion, and the antioxidant defense system was increased, suggesting that Nrf2 activation could be up-regulated. Interestingly, Kim et al.^10^ reported that BH extract 400 mg/kg intake reduced lipid peroxidation and increased antioxidant defense system similar to metformin 250 mg/kg intake, which confirms the excellent beneficial results of Korean BH intake. The present study showed that BH extract intake by 200 mg/kg showed the same results, confirming its competitiveness in terms of drug and economic efficiency.

We confirmed that Nrf2 activation was induced during AMPK phosphorylation and the occurrence of AMPK phosphorylation by BH extract ingestion is thought to be activated by Nrf2 by increased antioxidant enzyme expression. Thus, it is predicted that BH extract intake affects liver steatosis changes followed by a high-fat diet. These predictions were made in a previous study^10^, with increased antioxidant enzyme expression and hepatic tissue AMPK phosphorylation after BH extract ingestion, and our results were similar to those of previous studies.

In conclusion, we investigated the effects of a high-fat diet on obesity risk factors, NAFLD risk factors, and antioxidant enzymes when BH extract was used in mild diabetic obese mice and directly compared these effects with a metformin 250 mg/kg treatment as an AMPK activator. BH extract ingestion for 12 weeks improved lipid metabolism, lipid contents, and liver enzymes. The decrease in lipid peroxidation and the increase in the antioxidant defense system were also observed to be dose-dependent according to BH extract intake. These results are mediated through AMPK up-regulation in BH extract ingestion. The change in lipid metabolism gene expression was observed in adipose tissue through increased AMPK phosphorylation in hepatic tissue, suggesting that the hypothesis of this study is reasonable. Specifically, the results shown in the 200 mg/kg of Korean BH extract treatment were similar to that of metformin 250 mg/kg treatment and showed the possibility of competitive economic efficiency and comparable effects indicated in the previous study.

## References

[1] Wong VW, Chan WK, Chitturi S, Chawla Y, Dan YY, Duseja A, Fan J, Goh KL, Hamaguchi M, Hashimoto E, Kim SU, Lesmana LA, Lin YC, Liu CJ, Ni YH, Sollano J, Wong SK, Wong GL, Chan HL, Farrell G (2018). Asia-Pacific Working Party on Non-alcoholic Fatty Liver Disease guidelines 2017-Part 1: Definition, risk factors and assessment. *J Gastroenterol Hepatol*.

[2] Sattar N, Forrest E, Preiss D (2014). Non-alcoholic fatty liver disease. *BMJ*.

[3] Furukawa S, Fujita T, Shimabukuro M, Iwaki M, Yamada Y, Nakajima Y, Nakayama O, Makishima M, Matsuda M, Shimomura I (2004). Increased oxidative stress in obesity and its impact on metabolic syndrome. *J Clin Invest*.

[4] Videla LA, Rodrigo R, Orellana M, Fernandez V, Tapia G, Quinones L, Varela N, Contreras J, Lazarte R, Csendes A, Rojas J, Maluenda F, Burdiles P, Diaz JC, Smok G, Thielemann L, Poniachik J (2004). Oxidative stress-related parameters in the liver of non-alcoholic fatty liver disease patients. *Clin Sci (Lond)*.

[5] Szajdek A, Borowska EJ (2008). Bioactive compounds and health-promoting properties of berry fruits: a review. *Plant Foods Hum Nutr*.

[6] Raudsepp P, Anton D, Roasto M, Meremäe K, Pedastsaar P, Mäesaar M, Raal A, Laikoja K, Püssa T (2013). The antioxidative and antimicrobial properties of the blue honeysuckle (Lonicera caerulea L.), Siberian rhubarb (Rheum rhaponticum L.) and some other plants, compared to ascorbic acid and sodium nitrite. *Food Control*.

[7] Celli GB, Ghanem A, Brooks MSL (2014). Haskap berries (Lonicera caerulea L.)—A critical review of antioxidant capacity and health-related studies for potential value-added products. *Food Bioproc Tech*.

[8] Palikova I, Valentova K, Oborna I, Ulrichova J (2009). Protectivity of blue honeysuckle extract against oxidative human endothelial cells and rat hepatocyte damage. *J Agric Food Chem*.

[9] Wu S, Yano S, Chen J, Hisanaga A, Sakao K, He X, He J, Hou DX (2017). Berry Inhibit LPS-Induced Inflammation through Dual Modulation of Inflammatory and Antioxidant Mediators. *J Agri Food Chem*.

[10] Kim JW, Lee YS, Seol DJ, Cho IJ, Ku SK, Choi JS, Lee HJ (2018). Anti-obesity and fatty liver-preventing activities of Lonicera caerulea in high-fat diet-fed mice. *Int J Mol Med*.

[11] Hummer KE (2006). Blue honeysuckle: A new berry crop for North America. *J Am Pomol Soc*.

[12] Lee YS, Cho IJ, Kim JW, Lee SK, Ku SK, Lee HJ (2018). Evaluation of in vitro anti-oxidant and anti-inflammatory activities of Korean and Chinese Lonicera caerulea. *Nutr Res Pract*.

[13] Jung YM, Lee SH, Lee DS, You MJ, Chung IK, Cheon WH, Kwon YS, Lee YJ, Ku SK (2011). Fermented garlic protects diabetic, obese mice when fed a high-fat diet by antioxidant effects. *Nutrition Research*.

[14] Kim CM, Yi SJ, Cho IJ, Ku SK (2013). Red-koji fermented red ginseng ameliorates high fat diet-induced metabolic disorders in mice. *Nutrients*.

[15] Folch J, Lees M, Sloane Stanley G (1957). A simple method for the isolation and purification of total lipides from animal tissues. *J Biol Chem*.

[16] Kavutcu M, Canbolat O, Öztürk S, Olcay E, Ulutepe S, Ekinci C, Gökhun IH, Durak I (1996). Reduced enzymatic antioxidant defense mechanism in kidney tissues from gentamicin-treated guinea pigs: effects of vitamins E and C. *Nephron*.

[17] Lowry OH, Rosebrough NJ, Farr AL, Randall RJ (1951). Protein measurement with the Folin phenol reagent. *J Biol Chem*.

[18] Sun Y, Oberley LW, Li Y (1988). A simple method for clinical assay of superoxide dismutase. *Clinical Chemistry*.

[19] Kang SJ, Lee JE, Lee EK, Jung DH, Song CH, Park SJ, Choi SH, Han CH, Ku SK, Lee YJ (2014). Fermentation with Aquilariae Lignum enhances the anti-diabetic activity of green tea in type II diabetic db/db mouse. *Nutrients*.

[20] Lee JE, Kang SJ, Choi SH, Song CH, Lee YJ, Ku SK (2015). Fermentation of Green Tea with 2% Aquilariae lignum Increases the Anti-Diabetic Activity of Green Tea Aqueous Extracts in the High Fat-Fed Mouse. *Nutrients*.

[21] Yki-Järvinen H (2014). Non-alcoholic fatty liver disease as a cause and a consequence of metabolic syndrome. *Lancet Diabetes Endocrinol*.

[22] Sung KC, Jeong WS, Wild SH, Byrne CD (2012). Combined influence of insulin resistance, overweight/obesity, and fatty liver as risk factors for type 2 diabetes. Diabetes Care.

[23] Loomba R, Abraham M, Unalp A, Wilson L, Lavine J, Doo E, Bass NM (2012). Association between diabetes, family history of diabetes, and risk of nonalcoholic steatohepatitis and fibrosis. *Hepatology*.

[24] Chaovanalikit A, Thompson MM, Wrolstad RE (2004). Characterization and quantification of anthocyanins and polyphenolics in blue honeysuckle (Lonicera caerulea L.). *J Agri Food Chem*.

[25] Rupasinghe HV, Arumuggam N, Amararathna M, De Silva A (2018). The potential health benefits of haskap (Lonicera caerulea L.): Role of cyanidin-3-O-glucoside. *J Funct Foods*.

[26] Wei X, Wang D, Yang Y, Xia M, Li D, Li G, Zhu Y, Xiao Y, Ling W (2011). Cyanidin-3-O-β-glucoside improves obesity and triglyceride metabolism in KK-Ay mice by regulating lipoprotein lipase activity. *J Sci Food Agric*.

[27] Guo H, Ling W, Wang Q, Liu C, Hu Y, Xia M (2008). Cyanidin 3-glucoside protects 3T3-L1 adipocytes against H_2_O_2_-or TNF-α-induced insulin resistance by inhibiting c-Jun NH2-terminal kinase activation. *Biochem Pharmacol*.

